# Antiarthritic and Antioxidant Activities of *Antrocaryon micraster* Seed Extract and Its Fractions

**DOI:** 10.1155/2024/8838626

**Published:** 2024-01-27

**Authors:** Emmanuel Kofi Kumatia, Prince Kyei Baffour, Peter Bolah

**Affiliations:** ^1^Department of Phytochemistry, Centre for Plant Medicine Research, Mampong, Akuapem, Ghana; ^2^Department of Quality Management, Centre for Plant Medicine Research, Mampong, Akuapem, Ghana

## Abstract

Rheumatoid arthritis (RA) is an incurable debilitating disease which attacks the joints and impairs quality of life. *Antrocaryon micraster* is used to treat RA in African traditional medicine. However, its antiarthritic activity has not been pharmacologically studied. This study, therefore, reports the antiarthritic and antioxidant activities of *A. micraster* seed extract and its fractions. The seed extract (ASE) was produced by Soxhlet extraction and partitioned into petroleum ether (ASEP), ethyl acetate (ASEE), and aqueous (ASEA) fractions. The total polyphenolic content, DPPH antioxidant activity, and in vitro arthritic activity using the protein denaturation assay were evaluated for ASE and its fractions. The arthritic activity of the crude extract (ASE) and its most effective fraction (ASEA), in the in vitro assay, were then evaluated against CFA-induced arthritis in rats. The polyphenolic constituent of ASE was estimated to be 13.00 ± 0.00 mg/100 mg of GAE. ASEA contained the highest quantity of polyphenolic constituents (10.76 ± 0.00 mg/100 mg of GAE) among the fractions of the extract. ASE and ASEA produced profound antioxidant activity (IC_50_ = 20.17 ± 1.291 and 19.35 ± 0.865, respectively) which were similar to that of ascorbic acid (IC_50_ = 17.35 ± 0.500) in the DPPH free radical scavenging assay. Furthermore, in vitro antiarthritic activity of ASEA was 13.63 and 5.75 times higher than the antiarthritic activity of the crude extract and diclofenac sodium, respectively. In the CFA-induced arthritis assay, both ASE and ASEA significantly (*P* < 0.001) inhibited cachexia, paw edema, infiltration of inflammatory cells, pannus formation, and synovium damage. These results indicate that *A. micraster* seed extract and its fractions possessed significant antiarthritic activity via inhibition of oxidative stress, inflammation, protein denaturation, infiltration of inflammatory cells, and synovium injury due to its constituents such as polyphenols and phytosterols.

## 1. Introduction

The commonest source of medicine for the treatment of diseases in most developing counties is medicinal plants [1]. Furthermore, finished herbal products and herbal medicines have been incorporated into the orthodox health care delivery systems in countries such as China, India, and Ghana. The World Health Organization has reported that about 80% of the world population uses traditional medicine [2]. This may be due to the fact that medicinal plants are readily available and accessible at virtually no cost. Additionally, medicinal herbs and their products are perceived to be safer compared to orthodox medicines. Also, most people believe in the healing properties of medicinal herbs used in their localities. Hence, they are unwilling to experiment with unknown orthodox drugs.

Rheumatoid arthritis (RA) is a chronic systemic autoimmune disease which manifest in the form of general inflammation of the joints with pain, obliteration of articular tissues, and deformities in the joints with heightened probability of bone injury and cartilage devastation thereby producing extensive deformity [3]. RA has no cure. Hence, the current management strategy focuses on reduction of symptoms at the joints and to slow down its progression to disability [4].

Apart from the used of pharmaceutical drugs such as disease-modifying antirheumatic drugs (DMARDs) and nonsteroidal anti-inflammatory drugs (NSAIDs) in the management of RA, many medicinal plants and herbal preparations are also employed to treat arthritis. *Antrocaryon micraster* (A. Chev. and Guillaumin) is one of these plants. It is a large tropical rain forest tree of the Anacardiaceae or cashew family, which shed its leaf annually. The trunk of the plant is upright and cylindrical and can spread up to 1.5 m in diameter and 50 m tall [5]. The root, stem bark, leaf, fruit, and seed of the tree are used as herbal treatments against diseases such as pain and arthritis, cough, chest pain, stomachache, toothache, chicken pox, headache, inflammation, and boils and malaria [1, 5–8]. Although *A. micraster* is used to treat arthritis, the antiarthritic activity of the plant has not been pharmacologically evaluated. However, the antimalaria and antitrypanosomal activities of the plant have been studied [1, 9]. The aim of this study is therefore to evaluate the antiarthritic and antioxidant activities of *A. micraster* seed extract and its fractions using in vitro and in vivo models.

## 2. Materials and Methods

### 2.1. Chemicals and Reagents

Diclofenac sodium and Complete Freund's Adjuvant were acquired from Sigma Chemical Co. (St. Louis, USA). Petroleum ether (40–60°C) and ethyl acetate were supplied by Fisher Scientific (Loughborough, UK). Ethanol (99%) was obtained from Midland Ghana Limited (Tema). Normal saline was purchased from Otsuka Pharmaceuticals India Private Limited (Vasana-Chakawadi).

### 2.2. Sourcing of A. micraster Seed

The dried seed of *A. micraster* (AS) was purchased from a vendor at the GPRTU bus station in Koforidua (the capital town of the Eastern Region of Ghana) in February 2021. The identity of the seed was authenticated by Mr. Jonathan Dabo of the Forestry Research Institute of Ghana (FORIG), Kumasi. The seed was assigned a voucher specimen (FORIG 0015) and kept at the FORIG herbarium, as indicated in our previously published preprint of this manuscript [10].

### 2.3. Processing and Extraction

Sample of the seed was crushed into pieces and then milled into course powder. A total of 400 g of the AS course powder was extracted using the Soxhlet apparatus. Briefly, 200 g of AS was sequentially extracted with 1.5 L of 70% ethanol three times after every 4 hours. The second batch of 200 g was also treated with the same procedure. The extracts from the six extractions were combined and concentrated in vacuum (Eyeler N1110, Tokyo, Japan) at 46°C to 650 mL. Sample of the extract (400 mL) was transferred into a separating funnel and diluted with 100 mL of distilled water. It was then successively partitioned three times each with 500 mL petroleum ether, followed by 500 mL each of ethyl acetate three times. Each fraction was separately combined and dried in vacuum at 40°C. The aqueous fraction was also freeze-dried. The dried solids were labelled ASE, ASEP, ASEE, and ASEA which corresponds to the crude extract, petroleum ether, ethyl acetate, and aqueous fractions, respectively.

### 2.4. Animals and Ethical Considerations

The rats were kept in aluminum cages containing saw dust at 22°C±2°C and were fed on powdered commercial feed and sterilized water. The study was performed in compliance with the UK Animals (Scientific Procedures) Act 1986. The permission to perform the study was given by the Centre for Plant Medicine Research institutional review committee under 2010/63/EU animal experimental guidelines. All precautions were taken in order not to cause the animal undue pain or distress.

### 2.5. Qualitative Phytochemical Screening of the Extract

The phytochemical constituents present or absent in the extract were analyzed using methods described by Fong et al. [11].

### 2.6. Quantification of Polyphenols

The Folin–Ciocalteu method [12], with some modification, was employed to determine the total polyphenol content of the extract and the fractions. In summary, 2 mL of the Folin–Ciocalteu reagent (10%) was mixed with 0.4 mL of 1, 0.5, 0.25, 0.125, 0.0625, 0.03125, 0.015625, and 0.0078125 mg/mL each of the sample prior to addition of 1.6 mL and kept in the dark for 8 min. Thereafter, the mixture was added to 1.6 mL of 7.5% sodium carbonate solution in 15 mL test tubes and mixed and incubated again in the dark for 90 min. The absorbance was then measured at 765 nm using double beam UV/VIS spectrophotometer (BK-D590, Shandong, China). A standard calibration curve was also generated using gallic acid under similar experimental conditions from which the polyphenol concentrations in the extract and fractions were derived as gallic acid equivalent (GAE) per milligram of dry sample. The tests were done in triplicate.

### 2.7. Evaluation of Antioxidant Activity

The antioxidant activity of the extract and the fractions was evaluated by using DPPH free radical scavenging activity assay described by Kostyuk et al. [13]. Approximately, 4 mL of 0.2 mM, 2,2-diphenyl-1-picrylhydrazyl- (DPPH-) methanol solution was mixed with 2 mL each of 1, 0.5, 0.25, 0.125, 0.0625, 0.03125, 0.015625, or 0.0078125 mg/mL each of the test sample concentrations and incubated at room temperature (25°C) for 30 min in the dark. The absorbance of the mixture was measured at 517 nm with double beam UV/VIS spectrophotometer (BK-D590, Shandong, China). Ascorbic acid (Ac-A) and methanol were used as the standard drug and blank, respectively. The blank sample was prepared by mixing 2 mL of methanol in 0.2 mM of 2 mL DPPH solution. The tests were performed in triplicate. The % inhibition was estimated using the equation below.

(1) $$\begin{array}{c} \%\text{Inhibition}=\frac{\text{Absorbance of the blank}-\text{absorbance of test sample}}{\text{Absorbance of the blank}}\times 100\%. \end{array}$$

The % inhibition of the sample was then used to plot the log normalized response curve using GraphPad Prism 9.5.1 from which the antioxidant activity was estimated as the IC_50_ values.

### 2.8. In Vitro Protein Denaturation Assay

Protein denaturation assay was performed as described by Padmanabhan and Jangle [14], with slight modifications. Briefly, sodium diclofenac salt/extract concentrations of 3.90-2000 *μ*g/mL (2 mL) were each added to 0.2 mL of egg albumin from fresh egg and 2.8 mL of phosphate buffer saline (PBS, pH 6.4) and mixed thoroughly. The mixture was incubated at room temperature for 10 min and then heated at 70°C for 20 min and finally left to cool down. The turbidity of the mixture was read at an absorbance of 660 nm. The negative control consists of 10% DMSO in distilled water (*v*/*v*). The percentage inhibition of protein denaturation was calculated as

(2) $$\begin{array}{c} \%\text{Inhibition}=\frac{A\left(\text{NC}\right)-A\left(S\right)}{A\left(\text{NC}\right)}\times 100\%, \end{array}$$

where *A*(NC) is the absorbance of the negative control and *A*(*S*) is the absorbance of the sample.

GraphPad Prism was used to compute the 50% inhibitory concentration (IC_50_).

### 2.9. Acute Toxicity Test

Acute toxicity effect of ASE was tested in female SDR rats (*N* = 6) by adopting the method of Organization for Economic Co-operation and Development [15] with some amendments. A single dose of 5 g/kg of the extract was suspended in distilled water and mixed thoroughly to form a uniform solution without addition of any suspending agent since the extract was soluble in water. It was then administered to the rats using oral gavage at a rate of 10 mL/100 g body weight just once. The rats were then monitored at regular time intervals for indications of toxicity like paralysis, salivation, change in fur alignment, change in food and water intakes, lacrimation, unconsciousness, or death for 14 days.

### 2.10. Evaluation of Antiarthritic Activity against CFA-Induced Arthritis

Antiarthritic activity of the ASE and its fraction was evaluated by employing previously described methods [16, 17]. Briefly, arthritis was induced in the left hind paw of female Sprague Dawley rats (135-147 g) by intradermal injection of the left footpad with 0.2 mL Complete Freund's Adjuvant (CFA). The rats were grouped into 6 (*N* = 5). The crude seed extract (ASE), its aqueous fraction (ASEA), and the standard drug (D-Na) were dissolved in water to form a uniform solution. The solution was orally administered with gavage at 2.0 mL per rat daily for 14 consecutive days.

Group 1—ASE 25 mg/kg p.o.

Group 2—ASE 100 mg/kg p.o.

Group 3—ASEA 25 mg/kg p.o.

Group 4—ASEA 100 mg/kg p.o.

Group 5—D-Na 5 mg/kg p.o.

Group 6—2 mL of distilled water (CFA/arthritic control)

The baseline paw volume of the left hind paw was measured on day 0 (Vo) as the average of two measurements by a plethysmometer (Ugo Basile, Italy), 3 h prior to the induction of the arthritis.

Measurements of the paw volumes of the left hind limbs of each rat were repeated at 2-day intervals (Vt) from day 0 to day 28. The administration of the extracts and the standard drug was started on the 14th day after the arthritis induction. The change in paw volume which was taken as edema was calculated as (Vt − Vo).

### 2.11. Body Weight Measurement

The rats were weighed (Wo) before the commencement of the experiment. They were again weighed after every two days (Wt) until the 28th day. The mean change in the body weight of the rats was then calculated as shown below.

(3) $$\begin{array}{c} \text{Mean change in body weight}=\frac{\sum \left(\text{Weight of animal at Wt}-\text{Weight of animal at Wo}\right)}{\text{Total number of mice in a group}}. \end{array}$$

### 2.12. Histopathological Examination of the Ankle Joints

The process was executed as described by Patil et al. [18]. On the 29th day, the rats were euthanized. The arthritic ankle joints were removed and preserved in buffered formalin (10%) for 14 days. They were decalcified in 5% formic acid, fixed in paraffin, and split into 5 *μ* sections. The sections were stained with hematoxylin-eosin solution and analyzed under light microscope (Carl Zeiss, Primos Star, Germany) at 10x magnification for indicators of arthritis such as inflammatory cell infiltration, congested interstitial space, and development of pannus.

### 2.13. Statistical Analysis

GraphPad Prism version 6 statistical soft was employed for the data analysis, and the results are written as mean ± SEM. Variations were ascertained by comparative analysis using one-way or two-way analysis of variance (ANOVA), followed by Dunnett's or Tukey's multiple comparison test where applicable. Statistically significant was attained *P* < 0.05.

## 3. Results

### 3.1. Phytochemical Analysis of the Extract

The extract (ASE) contained phytoconstituents such as phytosterols, polyphenols, and free reducing sugars. Flavonoids, alkaloids, polyuronides, cyanogenic glycosides, triterpenes, saponins, and antracenosides were absent.

### 3.2. Yield and Polyphenol Contents of the Extract and Its Fractions

The yields of the extract and its fractions and their polyphenol contents are shown in Table 1. The yield and polyphenol content of the seed extract and its fractions were moderately low.

### 3.3. In Vitro Antioxidant Activity

The results of the IC_50_s obtained for ASE and its fractions in the DPPH assay are presented in Table 2.

ASE, ASEA, and the standard compound, ascorbic acid (Ac-A), gave similar IC_50_ values against the inhibition of free radical formation from DPPH. The IC_50_s of ASEP and ASEE were 4 and 5.4 times higher than that of Ac-A.

### 3.4. Protein Denaturation

The result of the in vitro antiarthritic activity measurement in the protein denaturation test is shown in Table 3.

ASE and all of its fractions demonstrated very good antiarthritic activity with very low IC_50_ values. However, ASEA produced the lowest IC_50_ (Table 3).

### 3.5. Acute Toxicity

None of the rats given the extract died or show any sign of toxicity within the 14-day observational period.

### 3.6. Effect of Treatment of ASE and ASEA on Body Weight of Arthritic Rats

The effect of the treatment of ASE, its aqueous fraction, and D-Na on the body weight alteration during arthritis is shown in Table 4.

The arthritic rats experienced weight lost (−8.63 ± 3.76 g) in the last 14 days. However, treatment of ASE and ASEA dose dependently and significantly (*P* < 0.05-0.001) increased their body weights. In the same manner, D-Na 5 mg/kg p.o. also significantly (*P* < 0.01) increased the body weight of the rats (Table 4).

### 3.7. Effect of ASE and ASEA on Edema Inhibition during CFA-Induced Arthritis

**Figure 1:**
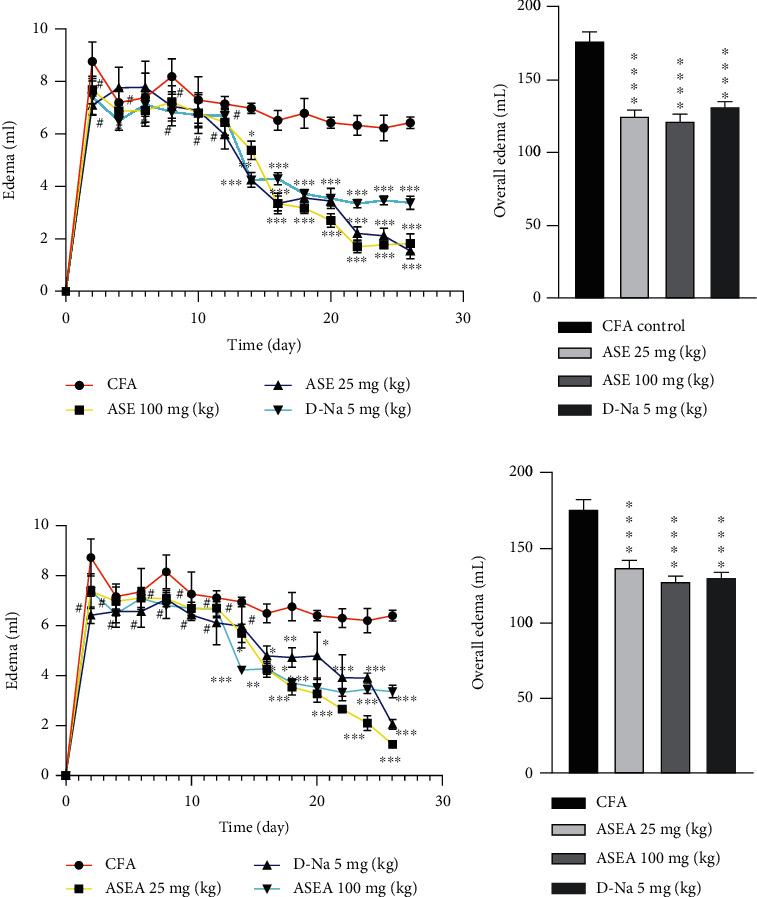
Effect of ASE and ASEA on edema formation on the (a, c) time course curve and (b, d) overall edema calculated as the area under the curve, during CFA-induced arthritis in rats' paw. Each point represents mean ± SEM. ^∗^*P* < 0.05, ^∗∗^*P* < 0.01, ^∗∗∗^*P* < 0.001, ^∗∗∗∗^*P* < 0.0001, or ^#^*P* > 0.05 compared to CFA-control rats.

The results of edema inhibition of ASE and ASEA in CFA-induced arthritis are shown in Figure 1.

ASE and ASEA reduced edema formation during CFA-induced arthritis in rats. The activity became statistically significant on days 14-28 on the time coursed curve after the commencement of treatment (Figures 1(a) and 1(c)). The overall edema inhibition was also statistically significant (*P* < 0.0001) and dose dependently for both ASE and ASEA (Figures 1(b) and 1(d)).

### 3.8. Histopathology of the Arthritic Ankle Joints

**Figure 2:**
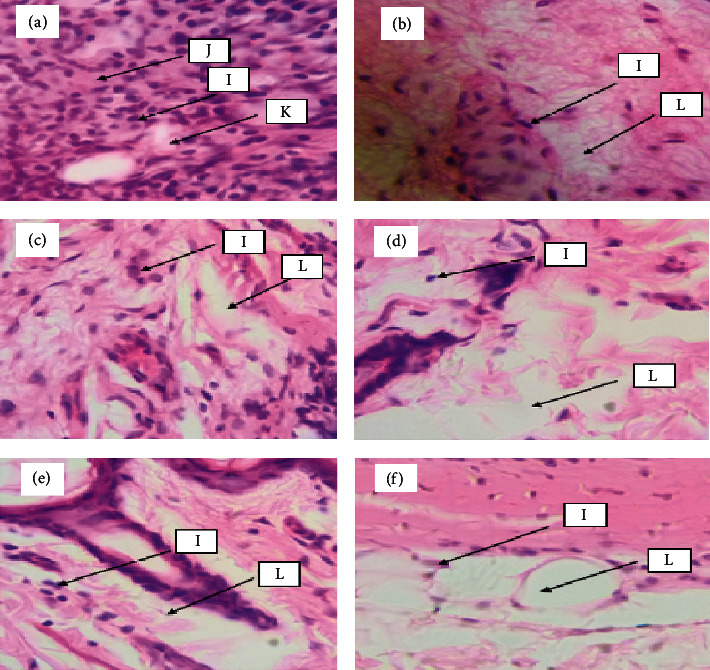
(a) Arthritic control; (b) DF; (c) ASE 25 mg/kg p.o.; (d) ASE 100 mg/kg p.o.; (e) ASEA 25 mg/kg p.o.; (f) ASEA 100 mg/kg p.o.: (I) inflammatory cells; (J) congested interstitial space; (K) pannus; (L) clear interstitial space.

The results of the histopathological assessment of the arthritic joints after termination are shown in Figures 2(a)–2(e).

It can be seen from the micrographs that there is synovial hyperplasia, disintegration of cartilage, wearing away of bone, and development of pannus which are very much pronounced in the nontreated arthritic control rats (Figure 2(a)) as compared to the ASE, ASEA, and D-Na-treated rats (Figure 2(a)).

## 4. Discussion

The yield and polyphenol content of 4.54% *w*/*w* and 13.00 ± 0.00 mg/100 mg of GAE obtained, respectively, for the Soxhlet extract of *A. micraster* seed were quite low, especially when compared to the yield and polyphenol content of 20.65% *w*/*w* and 234.960 ± 0.026 mg/g of GAE, respectively, reported for the stem bark of the same plant extracted by cold maceration [1], a method which gives a poorer yield than the Soxhlet extraction. The poor yield of the seed is due to its extreme hard nature. Surprisingly, also, the polyphenol content of the fractions of the extract (ASE) is inversely proportional to their yields (Table 1). Thus, when the fractions of ASE are in the order of increasing yield as ASEA < ASEP < ASEE, the reverse order of ASEE < ASEP < ASEA holds for the polyphenol content.

It had been reported that generation of autoantigens in some inflammatory or arthritic conditions could be as a result of denaturation of proteins in the affected tissue [19, 20]. Furthermore, it was shown that medicinal plant extracts which inhibit protein denaturation in vitro mostly demonstrate in vivo anti-inflammatory activity [21]. Moreover, the egg albumen protein denaturation test is very cheap and easy to perform and required less resources as compared to an in vivo animal study. The egg albumen protein denaturation test was, therefore, employed in this study to screen ASE and its fractions (ASEP, ASEE, and ASEA) in order to assess their anti-inflammatory activity which was an indication of their antiarthritic activity. The results show that the extract and its fractions produced very low 1C_50_ values which indicate their effective anti-inflammatory action. The in vitro anti-inflammatory activity of the test agents can be arranged as follows: ASEE < ASP < ASE < D-Na < ASEA. In real terms, D-Na, the stand drug, is only 2.4, 1.96, or 3.93 times more potent than ASE, ASP, or ASEE. However, the aqueous fraction of the extract, ASEA, was 5.75 more potent than D-Na and the most potent fraction among the three. The result also shows that although all the three fractions of ASE were active, ASEA and ASEP were more active than the parent extract, ASE. This shows that the most active anti-inflammatory constituents of ASE are both very polar compounds which resides in the aqueous fraction followed by very nonpolar ones in the petroleum ether fraction. It also shows that anti-inflammatory action of the active constituents of the extract was blocked by the other constituents when the fractions were together as one whole extract (ASE).

Since the crude extract is what is used to treat diseases in traditional medicine, it was therefore selected with ASEA for the in vivo antiarthritic activity test.

IC_50_ is defined as the minimum concentration of a substance required to produce 50% inhibition of the oxidation of DPPH molecule. Hence, the higher the IC_50_ value of the substance, the larger the amount required to prevent the oxidation and less effective the substance is. On the other hand, if the IC_50_ value is small, then little concentration of the substance is required to inhibit the oxidation of the DPPH molecule. And hence, more effective is the substance. In the DPPH free radical scavenging activity assay (Table 2), similar IC_50_ values were obtained for ASE, ASEA, and the standard compound, Ac-A, whereas the IC_50_s of ASEP and ASEE were 4 and 5.4 times higher than that of Ac-A. This indicates that ASE and ASEA possessed strong antioxidant activity similar to that of ascorbic acid (Ac-A). But ASEP and ASEE exhibited moderate antioxidant activity. It also indicates that the antioxidant constituents of *A. micraster* seed are more concentrated in its aqueous fraction than the petroleum ether and ethyl acetate fractions. Radical scavenging properties of substances are very vital in the prevention of the damaging effects of excess free radicals especially in chronic disease conditions such as diabetes, hypertension, cancers, and arthritis. Antioxidants such as polyphenols and tocopherols prevent these damages by donation of their hydrogen atoms [22, 23]. It has also been reported that free radical scavenging antioxidant activity of plant extracts is vastly associated with their total phenolic contents [22]. Therefore, the fraction of the extract (ASEA) which contains the higher quantity of polyphenols (Table 2) also demonstrated the most effective antioxidant activity in the DPPH assay. Thus, the antioxidant and antiarthritic activity of *A. micraster* seed extract and its fraction is due to its polyphenol and phytosterol constituents as shown by the qualitative and quantitative phytochemical tests.

Studies have shown that an effective in vitro DPPH free radical scavenging capacity of a plant extract indicates a corresponding in vivo antioxidant potency [24, 25]. Therefore, ASE and ASEA may also be very effective antioxidant agents in vivo which contributes to its antiarthritic activity.

Paw swelling is one of the classical signs of CFA-induced arthritis. And it can be easily measured using simple instruments thereby a quick procedure for the assessment of efficacy of antiarthitic agents [26]. The paw swelling is a result of cellular infiltration of the inflamed region, vascular permeability, and increase in fluid exudation [27]. An effective antiarthritic agent is therefore expected to significantly inhibit the causes of paw edema with corresponding decrease in paw swelling. Our results show that ASE and ASEA are very effective antiarthritic agent since they significantly (*P* < 0.0001) inhibited paw edema formation of their treatment groups (Figures 1(a)–1(d)).

Rheumatoid arthritis assaults the synovial membrane and causes the inflammation of the joint which is visible by histopathological examination [28]. It has also been reported that the release of NF-*κ*B and proinflammatory cytokines during rheumatoid arthritis leads to the classical symptoms such as synovial hyperplasia, pannus formation, and joint and bone disintegration in addition to the infiltration of the synovium by inflammatory cells which then destroys the synovial membrane [29, 30]. Hence, the histopathological examination of the joints was performed to assess the treatment outcomes on these symptoms. The results from our study indicated synovial hyperplasia, disintegration of cartilage, wearing away of bone, congested interstitial space, and development of pannus in the arthritic joints of the nontreated arthritic control rats (Figure 2(a)). On the other hand, no pannus development, very few inflammatory cells, clear interstitial spaces, and intact synovial membranes were observed in the ASE and ASEA and D-Na treatment groups (Figures 2(b)–2(f)). These results confirmed that *A. micraster* seed extract possessed antiarthritic activity by its ability to inhibit the damaging effects of proinflammatory cytokines on the joints.

Another classical symptom of rheumatoid arthritis is wasting away, a condition known as cachexia. Rheumatoid cachexia has been reported to be due to inflammation as a result of inability of the gut to absorb nutrients from food which is reserved by the intake of anti-inflammatory drugs [31, 32]. The results show that administration of *A. micraster* seed extract and its aqueous fraction significantly (*P* < 0.05) enhanced weight gain dose dependently compared to the arthritic control rats, confirming that the extract is an effective anti-inflammatory agent and hence restores nutrient absorption from food.

Also, the qualitative phytochemical screening indicates that the extract contains polyphenols and phytosterols. Further quantitative assay was then employed to calculate the amount of polyphenols in the extract. It was reported that polyphenols act on the inflammatory, oxidative, and apoptotic pathways to reduce the progression of RA. They also interfere with the inflammatory system through the MAPK track and NFATC1 in osteoblasts by modulation of MAPK, ILs 1 and 6, TNF-*α*, NF-*κ*B, JNK, extracellular signal-directed kinase (ERK1/2), activator protein-1 (AP-1), and COX-2 [33]. Moreover, a recent study also shows that phytosterols such as beta-sitosterol 3-palmatate and beta-sitosterol 3-myristate possessed significant antiarthritic activity at 3 *μ*mol/kg (p.o.) via inhibition of inflammation by 31.02 and 39.14%, respectively, against CFA-induced arthritis in rats which were equivalent to 30.79% anti-inflammatory activity of the standard and diclofenac sodium at the same dose [17]. Therefore, the presence of polyphenols and phytosterols in the extract and its fractions are responsible for its antiarthritis activity by acting on the inflammatory, oxidative, and apoptotic pathways of the CFA-induced arthritis.

Finally, since neither of the rats show any symptom of toxicity nor died during the acute toxicity test and the LD_50_ of the extract was above 5000 mg/kg p.o. per body weight, it indicates that the 70% Soxhlet ethanol extract of *A. micraster* seed (ASE) is safe for human consumption.

## 5. Conclusion

This is the first study to provide scientific validation of the use of *A. micraster* seed as a medicinal agent. The study has also shown that the 70% Soxhlet ethanol extract of *A. micraster* seed (ASE) and its petroleum ether (ASEP), ethyl acetate (ASEE), and aqueous (ASEA) fractions possessed considerable antiarthritic and antioxidant activities due to their polyphenolic and phytosterol contents. ASEA was the most active fraction among the three. The crude extract (ASE) and ASEA attenuate arthritis in rats through the inhibition of protein denaturation, inflammation, and cachexia and protection of the joints against adverse pathological alterations.

## Figures and Tables

**Table 1:** Weight, yields, and polyphenol contents of the ASE and its fractions.

Sample	Weight (g)	Yield (% *w*/*w*)	Polyphenol content (mg/100 mg of GAE)
ASE	18.15	4.54	13.00 ± 0.00
ASEP	3.86	1.45	1.70 ± 0.01
ASEE	5.04	1.89	0.40 ± 0.02
ASEA	3.20	1.20	10.76 ± 0.00

Abbreviations: ASE: 70% ethanol Soxhlet A. micraster seed extract; ASEP: petroleum ether fraction of ASE; ASEE: ethyl acetate fraction of ASE; ASEA: aqueous fraction of ASE.

**Table 2:** DPPH free radical scavenging activity of ASE and its fractions.

Sample	IC_50_ ± SEM (*μ*g/mL)
Ac-A	17.35 ± 0.500
ASE	20.17 ± 1.291
ASEP	69.35 ± 1.21
ASEE	93.22 ± 1.005
ASEA	19.35 ± 0.865

Abbreviations: ASE: 70% ethanol Soxhlet A. micraster seed extract; ASEP: petroleum ether fraction of ASE; ASEE: ethyl acetate fraction of ASE; ASEA: aqueous fraction of ASE.

**Table 3:** Summary of the IC_50_ values of sodium diclofenac, ASE, and its fractions against protein denaturation.

Sample	IC_50_ ± SEM (mg/mL)
D-Na	0.092 ± 0.00
ASE	0.218 ± 0.00
ASEP	0.180 ± 0.01
ASEE	0.362 ± 0.00
ASEA	0.016 ± 0.00

Abbreviations: ASE: 70% ethanol Soxhlet A. micraster seed extract; ASEP: petroleum ether fraction of ASE; ASEE: ethyl acetate fraction of ASE; ASEA: aqueous fraction of ASE.

**Table 4:** Mean change in body weight during the treatment period.

Sample	Mean change in body weight (g)
CFA-control	−8.63 ± 3.76
D-Na 10 mg/kg p.o.	10.93 ± 3.90^∗∗^
ASE 25 mg/kg p.o.	12.54 ± 3.12^∗∗∗^
ASE 100 mg/kg p.o.	19.29 ± 74.84^∗∗∗^
ASEA 25 mg/kg p.o.	10.07 ± 1.06^∗^
ASEA 100 mg/kg p.o.	16.14 ± 2.14^∗∗∗^

^∗^ *P* < 0.05, ^∗∗^*P* < 0.01, and ^∗∗∗^*P* < 0.001 compared to CFA-control where rats were treated with the substances in the last 14 days (days 14-28) out of the 28-day period of the experiment. Abbreviations: ASE: 70% ethanol Soxhlet *A. micraster* seed extract; ASEP: petroleum ether fraction of ASE; ASEE: ethyl acetate fraction of ASE; ASEA: aqueous fraction of ASE.

## Data Availability

The data upon which the conclusion in this article is based can be obtained from the corresponding author upon reasonable request.
